# A Cost–Utility Analysis of Two-Stage Screening Strategies Based on Waist-to-Height Ratio for Pediatric Metabolic Dysfunction-Associated Steatotic Liver Disease (MASLD) in China

**DOI:** 10.3390/healthcare14101343

**Published:** 2026-05-14

**Authors:** Yunfei Liu, Tianyu Huang, Jiajia Dang, Shan Cai, Jiaxin Li, Ruolan Yang, Jiabin Zhang, Kaiheng Zhu, Ziyue Sun, Yang Yang, Yajie Wang, Bo Xi, Yi Song

**Affiliations:** 1 Institute of Child and Adolescent Health, School of Public Health, Peking University, Beijing 100191, China; melatonin@pku.edu.cn (Y.L.); tyhuang@pku.edu.cn (T.H.); dangjj@bjmu.edu.cn (J.D.); 1710306207@pku.edu.cn (S.C.); lijiaxin@bjmu.edu.cn (J.L.); 2010306116@stu.pku.edu.cn (R.Y.); 2516397050@bjmu.edu.cn (K.Z.); sunziyue@bjmu.edu.cn (Z.S.); 2516395133@bjmu.edu.cn (Y.Y.); wang-yj@bjmu.edu.cn (Y.W.); 2 National Health Commission Key Laboratory of Reproductive Health, Peking University, Beijing 100191, China; 3 Peking University People’s Hospital, Beijing 100044, China; 2010301245@stu.pku.edu.cn; 4 School of Public Health, Shandong University, Jinan 250012, China; xibo2010@sdu.edu.cn

**Keywords:** metabolic dysfunction-associated steatotic liver disease, pediatric screening, waist-to-height ratio, cost-effectiveness analysis, lifestyle modification, Markov model

## Abstract

**Highlights:**

**What are the main findings?**

- Across willingness-to-pay (WTP) thresholds defined using either the national average gross domestic product (GDP) (WTP: $30,584.0 per QALY) or Beijing’s GDP (WTP $71,415.5 per QALY), waist-to-height ratio (WHtR)-based two-stage screening strategies for pediatric MASLD were consistently cost-effective.
- The optimal screening strategy varied by WTP level: WHtR combined with FibroScan^®^ was preferred at the WTP threshold based on the national average GDP, whereas WHtR combined with magnetic resonance imaging-proton density fat fraction (MRI-PDFF) became the optimal strategy under a higher WTP threshold based on Beijing’s GDP.

**What are the implications of the main findings?**

- These findings underscore the need to tailor pediatric MASLD screening policies to local resource availability, while supporting the broader implementation of early screening strategies to facilitate timely identification and intervention of pediatric MASLD.

**Abstract:**

**Background:** The prevalence of metabolic dysfunction-associated steatotic liver disease (MASLD) has increased rapidly in pediatric populations. Evidence on the cost-effectiveness of pediatric MASLD screening strategies remains limited. **Methods:** A decision tree combined with a Markov state-transition model was developed to evaluate the cost-effectiveness of three WHtR-based two-stage screening strategies among children aged 6–14 years in Beijing, China: WHtR combined with ultrasound (S1), WHtR combined with FibroScan^®^ (S2), and WHtR combined with magnetic resonance imaging-proton density fat fraction (MRI-PDFF) (S3), compared with no screening (S4). All screening strategies were combined with lifestyle modification programs, including dietary and exercise management. Model inputs were derived from the published literature, national survey data, and expert consensus. Costs and quality-adjusted life years (QALYs) were estimated from a healthcare system perspective over a 10-year time horizon, with a 3% annual discount rate. Incremental cost–utility ratios (ICURs) were calculated, and extensive one-way, two-way, and probabilistic sensitivity analyses were performed. **Results:** Our model indicated that, at a willingness-to-pay (WTP) threshold of $30,584.0 per QALY, corresponding to three times the gross domestic product (GDP) per capita of China, S2 was identified as the optimal strategy. At a higher WTP threshold of $71,415.5 per QALY, based on the GDP per capita of Beijing, S3 became the most cost-effective option. All three screening strategies were more cost-effective than no screening across both thresholds. Sensitivity analyses demonstrated that utility values for fibrosis stages and the response rate of the lifestyle modification program were the most influential parameters, and probabilistic sensitivity analysis confirmed the robustness of the baseline findings. **Conclusions:** To the best of our knowledge, this is the first cost-effectiveness analysis for pediatric MASLD in China. Model-based estimates suggest that early screening for MASLD in children using WHtR-based screening strategies is cost-effective, with FibroScan^®^ preferred in settings with average economic development and MRI-PDFF preferred in more affluent regions. These findings underscore the importance of context-specific implementation of early MASLD screening strategies in pediatric populations to mitigate long-term disease burden.

## 1. Introduction

Against the backdrop of the rapidly increasing prevalence of overweight and obesity among children [1], the burden of metabolic dysfunction-associated steatotic liver disease (MASLD), previously referred to as non-alcoholic fatty liver disease (NAFLD) [2], has also risen sharply in this population [3]. It is now recognized that MASLD confers an additional risk for other diseases, such as type 2 diabetes and cardiovascular disease [4,5], and is associated with a rapidly increasing risk of mortality [6]. Nevertheless, studies indicate that fewer than one-third of children with obesity undergo screening for fatty liver disease during outpatient visits, resulting in missed opportunities for early intervention and imposing a substantial social and economic burden [7]. The substantial burden of MASLD, combined with its insidious nature, underscores the urgent need to develop effective tools for early screening and intervention in children, aiming to reduce both disease burden and associated risks.

In hospital settings, several screening tools, such as abdominal ultrasound, FibroScan^®^ (Echosens, Paris, France) with controlled attenuation parameter (CAP), and magnetic resonance imaging-proton density fat fraction (MRI-PDFF), are commonly used, but they are not suitable for large-scale population screening. In contrast, in population-based settings, our previous work highlighted the potential of the waist-to-height ratio (WHtR) as a noninvasive indicator for identifying pediatric MASLD in China, given its simplicity and scalability [8]. However, its relatively low specificity limits its effectiveness when used alone, highlighting the need for stepwise screening strategies.

In practice, screening strategies involve not only diagnostic performance but also trade-offs between resource allocation, implementation feasibility, and long-term health benefits [9]. In settings with constrained healthcare resources, it is essential to identify screening strategies that maximize health gains relative to their costs. Health economic evaluation provides a systematic framework to compare strategies. Decision-analytic modeling approaches, such as decision trees and Markov models, enable the integration of disease progression, intervention effects, and long-term outcomes, allowing for the comparison of alternative screening strategies beyond short-term diagnostic accuracy [10]. While numerous studies have assessed the cost-effectiveness of screening strategies using these approaches among adults, evidence remains scarce for children [11,12,13].

To date, few studies have systematically evaluated the cost-effectiveness of pediatric MASLD screening strategies using a comprehensive decision-analytic framework. In particular, the comparative value of stepwise screening approaches that combine simple anthropometric indicators with imaging modalities remains unclear. In the current study, we established three WHtR-based two-stage screening strategies combined with lifestyle modification programs, including dietary and exercise management, and evaluated the cost-effectiveness of each strategy. We hypothesized that all three strategies would be cost-effective compared with no screening. We further hypothesized that one strategy would emerge as the most cost-effective, and that the optimal strategy might vary across different settings and willingness-to-pay (WTP) thresholds. Accordingly, the objective of this study was to evaluate the cost-effectiveness of three WHtR-based two-stage screening strategies for pediatric MASLD among children in Beijing, China, and to identify the optimal strategy.

## 2. Materials and Methods

We integrated data from multiple sources, including the published literature and large-scale population-based datasets, such as the Chinese National Survey on Students Constitution and Health (CNSSCH), and conducted analyses using a decision tree and Markov modeling framework. A two-round Delphi process was employed, during which 17 experts critically reviewed the model inputs and provided recommendations when additional or alternative data sources were available based on their expertise. We simulated a hypothetical cohort of 100,000 children aged 6–14 years in Beijing and evaluated the cost-effectiveness of different screening strategies from a healthcare system perspective. This manuscript was prepared and reported in accordance with the CHEERS 2022 checklist [14] (Table S1).

### 2.1. Model Structure

**Figure 1:**
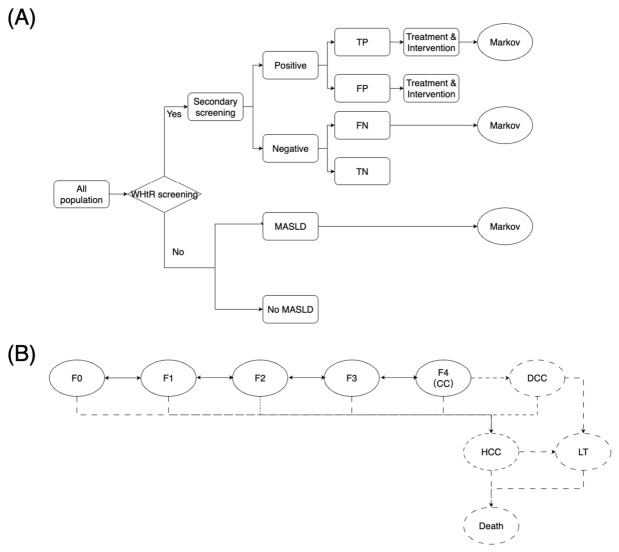
Decision-analytic modeling framework showing (**A**) the decision tree and (**B**) the Markov state-transition model. Note: The solid line represents the stages included in our study, while the dashed line represents the stages that exist in adults but were not included in this study. F0–F4 represent fibrosis stage 0 to fibrosis stage 4. MASLD, metabolic dysfunction-associated steatotic liver disease; WHtR, waist-to-height ratio; TP, true positive; FP, false positive; TN, true negative; FN, false negative; CC, cirrhosis; DCC, decompensated cirrhosis; HCC, hepatocellular carcinoma; LT, liver transplantation.

We implemented a decision-analytic model comprising an initial decision tree (Figure 1A) followed by a Markov state-transition model (Figure 1B) to reflect the short-term screening/diagnostic pathways and the long-term outcomes [10]. The decision tree captured one-off events surrounding screening, including test results and subsequent management, and assigned individuals to the corresponding post-screening health states that entered the Markov model [10].

In the decision tree, four strategies were compared: Strategy 1 (S1), WHtR combined with ultrasound; Strategy 2 (S2), WHtR combined with FibroScan^®^; Strategy 3 (S3), WHtR combined with MRI-PDFF; and Strategy 4 (S4), no screening. For the first three strategies, participants with a positive WHtR screening result (WHtR ≥ 0.48) proceeded to second-stage screening using ultrasound, FibroScan^®^, or MRI-PDFF, respectively. Participants with positive results at the second-stage screening subsequently received lifestyle modification programs, including dietary and exercise management.

In adult studies, Markov state-transition models typically include nine health states: no fibrosis (F0), mild fibrosis (F1), moderate fibrosis (F2), severe fibrosis (F3), compensated cirrhosis (CC), decompensated cirrhosis (DCC), hepatocellular carcinoma (HCC), liver transplantation (LT), and death [13]. Considering the extremely low prevalence of HCC among children aged 6–14 years in Beijing, and the fact that most pediatric HCC cases are attributable to hepatitis B virus rather than MASLD, the HCC and liver transplantation states were not included in our model [15]. We limited our health states to the first five stages. The Markov cycle length was set at 1 year, with a total time horizon of 10 years, and the state transitions were modeled using a life-table approach. In each cycle, individuals could remain in the same health state, progress to a more advanced disease state, or regress to a less severe state.

### 2.2. Delphi Process

We used a two-round Delphi process to review the plausibility of model inputs and to address key data gaps. A multidisciplinary expert panel was convened, covering child and adolescent health, epidemiology, hepatology, nutrition and physical activity, gastroenterology, endocrinology/metabolic disease, and portal hypertension. In total, 17 experts completed both rounds. Experts were asked to (i) assess the plausibility and clinical relevance of parameters identified from the literature, (ii) provide additional data sources to fill critical gaps, and (iii) provide input on appropriate sources for extrapolation when local pediatric data were unavailable.

Prior to the Delphi consultation, we conducted a structured evidence review to identify candidate parameters from multiple sources: (1) internal databases (e.g., CNSSCH), (2) publicly available datasets (e.g., National Health and Nutrition Examination Survey, NHANES), (3) the published literature, and (4) official yearbooks and reports. Parameters were collected across six domains: (a) sensitivity and specificity of screening tests, (b) pediatric MASLD prevalence and the baseline distribution of fibrosis stages, (c) annual transition probabilities under the natural history, (d) annual transition probabilities under lifestyle intervention, (e) costs, and (f) utilities. Based on these sources, a draft model and an initial parameter set were developed and iteratively refined through Delphi rounds.

For round 1, parameters were summarized and entered into an online questionnaire. Experts received a survey link and were asked to respond within one week; non-responders were reminded before the deadline. After round 1, we summarized agreement across expert ratings and incorporated feedback to refine the parameter set. The revised parameter set was then circulated in round 2 using the same procedure.

The expert authority coefficient (Cr) ranged from 0.55 to 0.76, indicating acceptable reliability. Kendall’s coefficients of concordance were 0.32 (*p* < 0.001) and 0.12 (*p* = 0.002) in rounds 1 and 2, respectively. The mean approval rate increased from 69.9% to 97.5%, suggesting high acceptance of the finalized parameter set.

### 2.3. Model Input

We used a WHtR ≥ 0.48 as the first-step screening tool, and the proportion of individuals who screened positive based on WHtR was estimated to be 22.6% using data from the CNSSCH 2019. Ultrasound (US score > 2), FibroScan^®^ (CAP ≥ 249 dB/m), and MRI-PDFF were applied as second-stage screening methods [16,17,18]. The sensitivity and specificity of each screening method in children are presented in Table 1, with parameter distributions specified based on previous studies [13].

Using a meta-analytic approach, we synthesized evidence from 13 studies conducted in China (details are provided in Table S2). Meta-regression analyses were performed with obesity prevalence obtained from CNSSCH as the independent variable to estimate the MASLD prevalence among children in Beijing across different age groups [19]. Specifically, a random-effects meta-regression model was used to account for between-study heterogeneity, and age-specific pooled prevalence estimates were derived and mapped to the corresponding age groups (6 to 14 years) in the modeled cohort. The initial distribution of fibrosis stages was derived from the NHANES population based on FibroScan^®^ assessments [20]. The initial proportion of different fibrosis stages is presented in Table 1.

**Table 1:** Test performance, initial distribution, cost and utilities.

Parameters	Value	Distribution	Age	Reference
**Test performance**				
SE for Ultrasound (US Score > 2)	0.52 (0.41, 0.64)	Beta	5 to 19	[16]
SP for Ultrasound (US Score > 2)	0.96 (0.91, 0.99)	Beta	5 to 19	[16]
SE for FibroScan^®^ (CAP ≥ 249 dB/m)	0.72 (0.64, 0.79)	Beta	4 to 17	[17]
SP for FibroScan^®^ (CAP ≥ 249 dB/m)	0.98 (0.97, 0.98)	Beta	4 to 17	[17]
SE for MRI-PDFF	0.95 (0.92, 0.97)	Beta	7 to 18	[18]
SP for MRI-PDFF	0.92 (0.77, 0.98)	Beta	7 to 18	[18]
**Initial distribution**				
Proportion of F0	88.5%		13 to 17	[20]
Proportion of F1	5.2%		13 to 17	[20]
Proportion of F2	3.5%		13 to 17	[20]
Proportion of F3	1.6%		13 to 17	[20]
Proportion of F4	1.2%		13 to 17	[20]
**Cost**				
Routine physical examination ($)	2.9		—	[21]
Ultrasound ($)	16.5		—	[21]
FibroScan^®^ ($)	13.0		—	Peking University People’s Hospital
MRI-PDFF ($)	87.0		—	[21]
Blood biochemical testing combination ($)	22.5		—	Peking University People’s Hospital
Fixed costs of lifestyle modification program ($)	10.5		6 to 14	[22,23]
Variable costs of lifestyle modification program ($)	21.7		6 to 14	[22,23]
**Utilities**				
F0	0.95 (0.93, 1.00)	Beta	≥18	[24]
F1	0.85 (0.79, 0.92)	Beta	≥20	[12]
F2	0.85 (0.79, 0.92)	Beta	≥20	[12]
F3	0.73 (0.64, 0.82)	Beta	≥18	[25]
F4	0.66 (0.49, 0.83)	Beta	≥18	[25]

Note: CAP, controlled attenuation parameter; MRI-PDFF, magnetic resonance imaging-proton density fat fraction; SE, sensitivity; SP, specificity. The proportion was derived from NHANES, and the proportions of F0 and F1 were further estimated from a Markov model given the transition probability. F0–F4 represent fibrosis stage 0 to fibrosis stage 4. Routine physical examination includes measurements of height, waist circumference, and blood pressure; blood biochemical testing combination includes measurements of triglycerides (TG), high-density lipoprotein (HDL), and fasting plasma glucose (FPG).

The age–sex-specific transition probabilities as well as the distributions were primarily obtained from Estes C’s and Younossi Z’s studies [26,27]. The detailed parameters are shown in Table 2. The treatment effect applied to the lifestyle modification group was set as 27.7%, based on estimates derived from the control group that received standard-of-care advice on diet and exercise in the Treatment of NAFLD in Children (TONIC) study, in which fibrosis was confirmed by liver biopsy [28]. Additionally, we assumed that the lifestyle modification program was effective in 40% of the population who received it [12].

The model incorporated costs related to routine physical examinations, including measurements of height, waist circumference, and blood pressure; imaging-based screening, including ultrasound, FibroScan^®^, and MRI-PDFF; and blood biochemical testing, including measurements of triglycerides (TG), high-density lipoprotein (HDL), and fasting plasma glucose (FPG). Cost estimates were obtained from the Beijing Physical Examination Center [21] and Peking University People’s Hospital. Costs were valued using provider fees, which are expected to incorporate capital, overhead and staffing costs; therefore, these components were not costed separately. The cost of the lifestyle modification program was estimated based on a cluster randomized controlled study conducted in Beijing in 2023 [23]. The costs were further divided into fixed and variable components based on the proportions calculated from Meng Li et al.’s study [22]. All utility values, as well as the distributions, were obtained from the literature [12,24,25]. All costs and utility parameters are provided in Table 1.

### 2.4. Statistical Analysis

The cost-effectiveness of strategies was evaluated using the incremental cost–utility ratio (ICUR), which is defined as follows:

(1) $$ICUR={(Cost}_{1}-{Cost}_{2})/{(Utility}_{1}-{Utility}_{2})$$

where ${Cost}_{1}\;and\;{Cost}_{2}$ represent the costs of two different strategies, and ${Utility}_{1}\;and\;{Utility}_{2}$ represent the corresponding utilities, measured in quality-adjusted life years (QALYs).

S4 was used as the reference strategy. We evaluated the cost-effectiveness of S1–S3 relative to S4 and conducted pairwise cost-effectiveness comparisons among S1–S3. A discount rate of 3% per year was applied to both costs and utilities. All costs were inflated to 2019 USD, and the willingness-to-pay (WTP) thresholds were set at $30,584.0 per QALY and $71,415.5 per QALY, corresponding to three times the gross domestic product (GDP) per capita of China and Beijing, respectively [29]. A screening strategy was considered cost-effective if its ICUR was less than or equal to the predefined WTP threshold.

### 2.5. Sensitivity Analysis

One-way sensitivity analyses were performed for all model parameters using the assigned distributions. In addition, several scenario analyses were conducted: (1) the discount rate was set at 0% and 5%; (2) the response rate of the lifestyle modification program was varied between 30% and 100%; (3) the response rate of the lifestyle modification program was reduced to 75% among individuals with advanced fibrosis (F3 and F4), reflecting limited mobility and reduced responsiveness to intervention [30]; and (4) adolescents aged 15–24 years were assumed to have higher disease progression rates and lower regression rates [31]. In addition, we conducted time-horizon sensitivity analyses using shorter horizons (1-year and 5-year) to assess the impact of the time horizon on the results.

Two-way sensitivity analyses were conducted on the sensitivity and specificity of each second-stage screening modality. In these analyses, we examined the conditions under which one screening strategy dominated another by varying only the sensitivity and specificity of a single strategy, thereby assessing the relative superiority between competing strategies. Probabilistic sensitivity analysis (PSA) was performed using Monte Carlo simulation with 1000 iterations, as informed by previous studies [12], simulating a cohort of 100,000 individuals by varying model input parameters according to their assigned probability distributions.

All analyses and data visualization were performed with R version 4.5.1.

## 3. Results

### 3.1. Baseline Analysis

**Figure 2:**
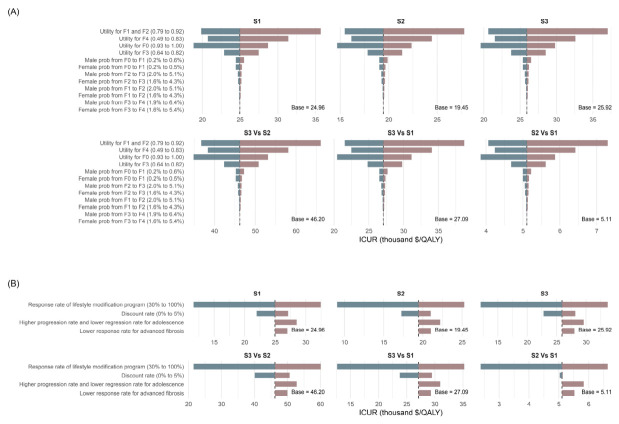
Tornado diagrams showing incremental cost–utility ratios for (**A**) key parameters and (**B**) alternative scenarios. Note: F0–F4 represent fibrosis stage 0 to fibrosis stage 4. S1, WHtR + Ultrasound strategy; S2, WHtR + FibroScan^®^ strategy; S3, WHtR + MRI-PDFF strategy; S4, no screening strategy; ICUR, incremental cost–utility ratios; MRI-PDFF, magnetic resonance imaging-proton density fat fraction. Blue indicates that the ICUR in that scenario is lower than in the base analysis, while red indicates that the ICUR is higher than in the base analysis. The results show that the results were most sensitive to fibrosis-related utilities and the response rate of lifestyle modification; S2 remained the most cost-effective strategy at national-based WTP thresholds, while S3 became optimal at higher thresholds.

All three screening strategies were cost-effective compared with no screening, with WHtR combined with FibroScan^®^ (S2) being the most cost-effective option at national-based WTP thresholds, while WHtR combined with MRI-PDFF (S3) became optimal at higher thresholds. The costs of WHtR combined with ultrasound (S1), S2, and S3 were $2.76 million, $2.98 million, and $5.24 million, with corresponding incremental QALYs of 110.6, 153.2, and 202.1, respectively. The ICURs were $24,960.5 per QALY, $19,445.7 per QALY and $25,924.2 per QALY, respectively, which indicated that all three strategies were more cost-effective than no screening (S4). Compared with S1, S2 would additionally cost $0.22 million, gain 42.6 QALY, with an ICUR of $5164.3 per QALY. Compared with S2, S3 would additionally cost $2.26 million, gain 48.9 QALY, with an ICUR of $46,216.8 per QALY. At a WTP of $30,584.0 per QALY and $71,415.5 per QALY, S2 and S3 were identified as the optimal options, respectively (Table 3, Figure 2).

### 3.2. One-Way Sensitivity Analysis

The results were most sensitive to fibrosis-related utilities and the response rate of lifestyle modification; S2 remained the most cost-effective strategy at national-based WTP thresholds, while S3 became optimal at higher thresholds. One-way sensitivity analysis indicated that the utility assigned to fibrosis stage F1 and F2 would be the most influential factor among all parameters. When the utility value was set at 0.92 and a WTP threshold at $30,584.0 per QALY, only S2 remained cost-effective compared with S4, with an ICUR of $27,791.3 per QALY. However, at a WTP threshold of $71,415.5 per QALY, all screening strategies remained cost-effective compared with S4, and S3 would be identified as the optimal option when utilities or transition probability varied (Figure 2A).

Across the scenario analyses, the response rate of the lifestyle modification program was the most influential factor affecting the ICURs among the different strategies. When the response rate was set at 30% and the WTP threshold at $30,584.0 per QALY, only S2 remained cost-effective compared with S4, with an ICUR of $25,276.1 per QALY. However, at a WTP threshold of $71,415.5 per QALY, all strategies remained cost-effective compared with S4, and S3 was identified as the optimal option under different scenarios (Figure 2B). In addition, none of the screening strategies (S1, S2, and S3) were cost-effective over shorter time horizons (Table S3), likely reflecting the higher upfront costs of screening and the delayed accrual of health benefits.

### 3.3. Two-Way Sensitivity Analysis

**Figure 3:**
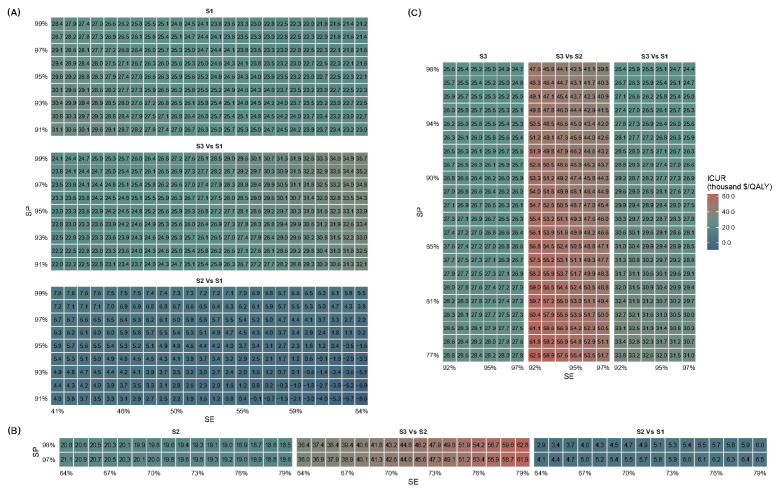
Dominance maps showing optimal strategies across sensitivity and specificity for (**A**) ultrasound, (**B**) FibroScan^®^, and (**C**) MRI-PDFF. Note: SE, sensitivity; SP, specificity; S1, WHtR + ultrasound strategy; S2, WHtR + FibroScan^®^ strategy; S3, WHtR + MRI-PDFF strategy; ICUR, incremental cost–utility ratios; MRI-PDFF, magnetic resonance imaging-proton density fat fraction. The results show that S2 consistently dominated S1, while S3 was optimal only at higher WTP thresholds or with high diagnostic performance.

S2 consistently dominated S1, while S3 was optimal only at higher WTP thresholds or with high diagnostic performance. For ultrasound-based screening, when sensitivity and specificity were 0.41 and 0.91, the ICURs of S1 versus S4, S3 versus S1, and S2 versus S1 were $31,137.2, $21,966.3, and $3982.8 per QALY, respectively. At a sensitivity and specificity of 0.64 and 0.99, the corresponding ICURs were $21,186.6, $35,705.3, and $5519.1 per QALY, respectively (Figure 3A). For FibroScan^®^-based screening, when sensitivity and specificity were 0.64 and 0.97, the ICURs of S2 versus S4, S3 versus S2, and S2 versus S1 were $9514.8, $35,984.5, and $4111.2 per QALY, respectively. As these parameters increased to 0.79 and 0.98, the ICUR of S2 versus S4 increased to $18,464.3 per QALY, while the ICURs of S3 versus S2 and S2 versus S1 rose to $62,757.5 and $4943.1 per QALY, respectively (Figure 3B). For MRI-PDFF-based screening, when sensitivity and specificity were 0.92 and 0.77, the ICURs of S3 versus S4, S2, and S1 were $28,803.6, $62,491.9, and $33,799.6 per QALY, respectively. At a sensitivity and specificity of 0.97 and 0.98, the corresponding ICURs declined to 24,687.1, 39,782.2, and 24,371.1 per QALY, respectively (Figure 3C).

At a WTP of $30,584.0 per QALY, when the sensitivity and specificity of ultrasound exceed 0.58 and 0.97, respectively, S3 no longer dominates S1; however, S2 consistently dominates S1. Regardless of variations in the sensitivity and specificity of FibroScan^®^, S3 does not dominate S2, whereas S2 always dominates S1. Similarly, irrespective of changes in the sensitivity and specificity of MRI-PDFF, S3 does not dominate S2; however, when its sensitivity and specificity exceed 0.93 and 0.85, respectively, S3 is able to dominate S1. At a WTP of $71,415.5 per QALY, however, regardless of variations in the sensitivity and specificity of ultrasound, FibroScan^®^, and MRI-PDFF, S3 consistently dominates both S2 and S1, while S2 consistently dominates S1.

### 3.4. Probabilistic Sensitivity Analysis

**Figure 4:**
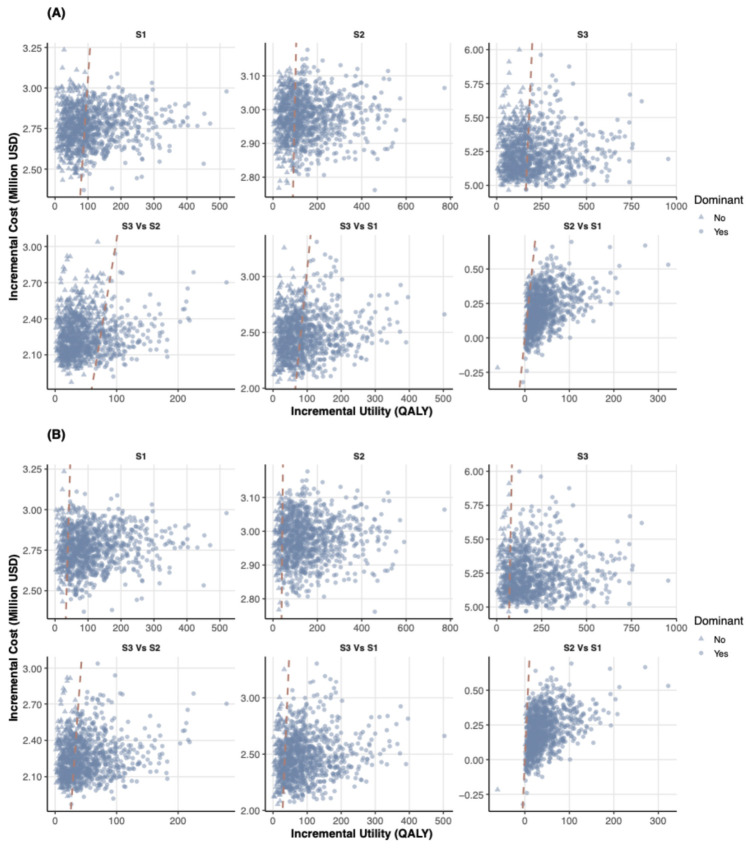
Probabilistic sensitivity analysis showing incremental costs and effects for (**A**) WTP = $30,584.0 per QALY and (**B**) WTP = $71,415.5 per QALY. Note: S1, WHtR + ultrasound strategy; S2, WHtR + FibroScan^®^ strategy; S3, WHtR + MRI-PDFF strategy; MRI-PDFF, magnetic resonance imaging-proton density fat fraction. The red dashed line indicates the threshold for cost-effectiveness at the given WTP. The results show that Screening was likely to be cost-effective across different WTP thresholds.

**Figure 5:**
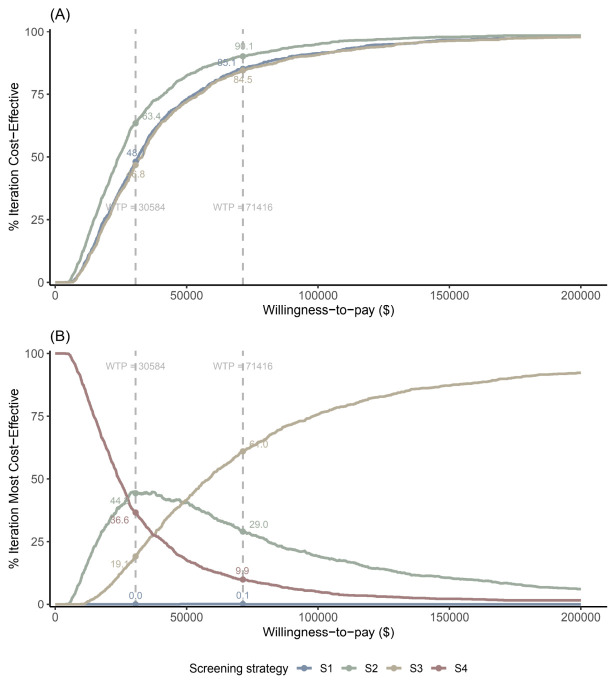
Cost-effectiveness acceptability curves showing strategy performance for (**A**) cost-effectiveness relative to S4 and (**B**) optimal strategy probabilities. Note: S1, WHtR + ultrasound strategy; S2, WHtR + FibroScan^®^ strategy; S3, WHtR + MRI-PDFF strategy; S4, no screening strategy; MRI-PDFF, magnetic resonance imaging-proton density fat fraction; WTP, willingness-to-pay. The results show that S2 had the highest probability of being cost-effective at national-based WTP thresholds, while S3 became the most cost-effective strategy at higher thresholds.

S2 had the highest probability of being cost-effective at national-based WTP thresholds, while S3 became the most cost-effective strategy at higher thresholds. The ICURs of S1, S2 and S3 compared with S4 were $31,572.7 per QALY (95% uncertainty interval [UI]: 8404.2, 176,773.6), $24,186.9 per QALY (6402.6, 135,999.1) and $33,094.1 per QALY (8627.2, 183,485.0). At a WTP of $30,584.0 per QALY, S1, S2, and S3 were more cost-effective than S4 in 48.1%, 63.4%, and 46.8% of iterations, respectively (Figure 4A and Figure 5A). At a WTP of $71,415.5 per QALY, S1, S2, and S3 were more cost-effective than S4 in 85.1%, 90.1%, and 84.5% of iterations, respectively (Figure 4B and Figure 5A).

As WTP increased, the proportion of iterations in which S4 was the most cost-effective strategy decreased, while that of S3 increased. The proportion for S2 initially increased and then decreased, reaching its peak of 44.9% at a WTP of $29,600 per QALY. At a WTP of $30,584.0 per QALY, S1, S2, S3, and S4 were the most cost-effective strategies in 0.0%, 44.3%, 19.1%, and 36.6% of iterations, respectively. At a WTP of $71,415.5/QALY, S1, S2, S3, and S4 were the most cost-effective strategies in 0.1%, 29.0%, 61.0%, and 9.9% of iterations, respectively (Figure 5B).

## 4. Discussion

This study is the first to evaluate the cost-effectiveness of MASLD screening in the pediatric population in China. Building on our previous work [8], in which a WHtR ≥ 0.48 was proposed as a practical indicator for identifying children who warrant further MASLD screening, we assessed the cost-effectiveness of three WHtR-based screening strategies: WHtR combined with ultrasound (S1), WHtR combined with FibroScan^®^ (S2), and WHtR combined with MRI-PDFF (S3). We further examined how the optimal screening strategy varied across different WTP thresholds. Under the modeled framework, when the WTP threshold was set at $30,584.0 per QALY, based on the national per capita GDP of China, S2 emerged as the optimal option. At a higher WTP threshold of $71,415.5 per QALY, derived from the per capita GDP of Beijing, S3 was identified as the optimal strategy. These findings were supported by sensitivity analyses. In addition, PSA demonstrated that as the WTP threshold increased, the strategy with the highest probability of being cost-effective transitioned from no screening (S4) to S2 and ultimately to S3.

In adult populations, several professional organizations have issued recommendations regarding MASLD screening. For example, the European Association for the Study of the Liver (EASL) and the Asian Pacific Association for the Study of the Liver (APASL) recommend targeted screening among individuals with obesity, metabolic syndrome, or diabetes [32,33]. In pediatric populations, existing guidance has also supported screening for MASLD. For instance, the American Association for the Study of Liver Diseases (AASLD) has recommended screening in children [34], a recent international multidisciplinary consensus recommended screening for MASLD in overweight and obese children [35], and the American Academy of Pediatrics has additionally suggested that factors such as central obesity should be considered in pediatric screening [7]. However, the cost-effectiveness of existing guidelines and recommendations has not been formally evaluated. Pediatric MASLD differs substantially from adult disease in terms of natural history, disease progression, and clinical management pathways, which may lead to different cost and health outcome trajectories [34]. Therefore, economic evidence derived from adult populations may not be directly applicable, underscoring the need for pediatric-specific economic evaluations to inform screening strategies. In the present study, we used the WHtR, a robust indicator of central obesity derived from height and waist circumference, which are universally collected in annual routine health examinations of primary and secondary school students in China [36]. We combined WHtR with commonly used screening modalities and evaluated the cost-effectiveness of these strategies. Our findings demonstrate the cost-effectiveness of WHtR-based screening strategies and underscore the importance of early and feasible screening for metabolic dysfunction-associated steatotic liver disease in pediatric populations.

Given the unique physiological and developmental characteristics of pediatric populations, there are currently no pharmacological therapies approved specifically for the treatment of MASLD or liver fibrosis in children. Lifestyle modification programs, including dietary and exercise management, remain the primary recommended approach [37]. Accumulating evidence indicates that dietary and exercise management are effective across diverse pediatric populations, with improvements in liver fibrosis largely mediated by weight reduction [38] and improvements in insulin sensitivity [39,40]. Notably, several studies have indicated that exercise may reduce the risk of MASLD independently of sustained weight loss [41], highlighting its metabolic benefits. In the present study, we used data from the control group receiving standard dietary and exercise management in the TONIC trial and applied the transition probability of 27.7% for the intervention group [28]. In addition, based on previous studies, we considered a response rate of 40% to be appropriate and examined the impact of different response rates on the results in sensitivity analyses [12]. Our findings indicate that response rate is one of the most influential factors of the ICUR, underscoring the critical importance of improving adherence and optimizing the efficacy of nutrition- and lifestyle-based managements. Furthermore, a substantial proportion of total costs was attributable to the lifestyle modification program in our model. Although these costs are relatively high at present, emerging evidence suggests that artificial intelligence-based intervention programs have been proven to be as effective as human-led intervention programs [42], indicating the potential for future reductions in the cost of the modification program.

A notable strength of our screening approach is its noninvasive nature, which is particularly advantageous in pediatric populations by reducing procedural burden and improving acceptability, thereby supporting early identification and broader implementation alongside nutrition- and lifestyle-based managements [34]. The specific context of Beijing warrants particular consideration, as its relatively high GDP per capita is associated with a higher WTP threshold. Although MRI-PDFF is not routinely used for MASLD screening because of its higher cost, its excellent sensitivity and specificity, quantitative assessment of hepatic steatosis, lack of operator dependence, and extensive validation in pediatric populations support the cost-effectiveness of S3 in this setting [34]. When extrapolating these findings to other provinces or regions, however, the application of lower WTP thresholds may be more appropriate, under which S2, combining WHtR screening with FibroScan^®^, emerges as the preferred option. This is particularly relevant in resource-limited settings, where constraints in healthcare resources and access to advanced imaging technologies may preclude the adoption of more costly strategies. In such contexts, S2 represents a cost-effective and scalable approach for public health implementation. Policy-makers should tailor screening strategies according to local resource availability, healthcare infrastructure, and population risk to maximize feasibility and health impact. S1, combining WHtR screening with ultrasound, remains cost-effective compared with S4. At WTP thresholds of $30,584.0 per QALY and $71,415.5 per QALY, the probabilities that S1 is more cost-effective than S4 were 48.1% and 85.1%, respectively. Nevertheless, the comparable cost and superior diagnostic performance of FibroScan^®^ limit the likelihood that S1 represents the optimal choice where FibroScan^®^ is available, although it may still serve as a pragmatic alternative in settings lacking access to FibroScan^®^ equipment.

Our study has several limitations. First, capital costs related to equipment acquisition were not explicitly itemized, and potential cost variations attributable to differences in staffing and clinical workflows across institutions were not separately modeled; instead, the analysis was limited to one-time costs associated with the implementation of screening. However, these cost structures may vary across regions and healthcare systems, which could influence the generalizability of the findings. In particular, in settings with lower examination or labor costs, the screening strategies evaluated may be more cost-effective than estimated in the present study. Second, the use of a 10-year time horizon may underestimate the long-term benefits of early screening, particularly given that MASLD is a chronic and progressive condition extending into adulthood. While this choice reflects a balance between capturing longer-term outcomes and minimizing strong assumptions regarding the transition from childhood to adulthood, it may limit the ability of the model to fully capture lifetime cost-effectiveness. Therefore, the present analysis should be regarded as a relatively conservative estimate of the potential long-term economic benefits of pediatric MASLD screening. Third, complications such as type 2 diabetes and cardiovascular disease were not incorporated into the model [6], which may result in an underestimation of utility losses. As a consequence, the ICUR may be overestimated, suggesting that the evaluated screening strategies could in fact be more cost-effective than reported. In addition, the lifestyle management assessed may provide broader health benefits beyond MASLD, including potential improvements in both physical and mental health outcomes among children [43]. Fourth, we did not explicitly model potential challenges associated with long-term lifestyle management, as the effectiveness of sustained lifestyle modification may diminish over time [44]. Fifth, many of the data inputs used in the model were derived from pediatric populations outside China, so caution is therefore required when generalizing these findings to Chinese populations. Sixth, several parameters, particularly utility values, were derived from adult populations, which may affect the generalizability of our findings to children and adolescents. Given the limited availability of pediatric utility data, these external inputs were used as approximations and may not fully reflect health-related quality-of-life trajectories in children with MASLD, potentially introducing additional uncertainty into our results. Seventh, parameter correlations were not modeled in the PSA, which may have influenced the results of our study. Finally, transition probabilities were largely informed by two published studies rather than estimated from original empirical data; however, additional sensitivity analyses were performed, and the results remained robust. Nevertheless, these relationships warrant further validation in future studies using original empirical data. In addition, the Delphi process used in parameter selection was intended to address current data limitations and should not be interpreted as a formal validation of the model structure or assumptions.

## 5. Conclusions

Our findings indicated that early screening for MASLD in children is cost-effective compared with no screening. In regions with economic development levels approximating the national average in China, screening based on WHtR combined with FibroScan^®^ represents the optimal strategy, whereas in more economically developed settings such as Beijing, WHtR combined with MRI-PDFF emerges as the preferred option. These findings highlight the importance of tailoring MASLD screening strategies to local resource constraints and economic contexts and support the implementation of WHtR-based MASLD screening strategies to reduce the long-term disease burden in pediatric populations across the life course. Future research should focus on piloting WHtR-based two-stage screening programs in school settings to assess real-world feasibility and effectiveness, given the urgent and increasing burden of pediatric obesity and MASLD globally.

## Figures and Tables

**Table 2:** Transition probabilities.

Parameters	Annual Transition Probabilities (Boys)	Annual Transition Probabilities (Girls)	Distribution	Age	Reference
F0 to F1	0.4% (0.2%, 0.6%)	0.3% (0.2%, 0.5%)	Beta	5 to 24	[26]
F1 to F0	6%	6%		5 to 24	[27]
F1 to F2	3.3% (2.0%, 5.1%)	2.8% (1.6%, 4.3%)	Beta	5 to 24	[26]
F2 to F1	6%	6%		5 to 24	[27]
F2 to F3	3.3% (2.0%, 5.1%)	2.8% (1.6%, 4.3%)	Beta	5 to 24	[26]
F3 to F2	6%	6%		5 to 24	[27]
F3 to F4	3.4% (1.9%, 6.4%)	2.8% (1.6%, 5.4%)	Beta	5 to 24	[26]
F4 to F3	6%	6%		5 to 24	[26]

Note: F0–F4 represent fibrosis stage 0 to fibrosis stage 4.

**Table 3:** Cost–utility of three strategies.

Strategy	Cost (Million $)	Utility (QALYs)	ICUR ($/QALYs)
S1	2.76	110.6	24,960.5
S2	2.98	153.2	19,445.7
S3	5.24	202.1	25,924.2
S4	0	0	Reference

Note: S1, WHtR + ultrasound strategy; S2, WHtR + FibroScan^®^ strategy; S3, WHtR + MRI-PDFF strategy; S4, no screening strategy; ICUR, incremental cost–utility ratios.

## Data Availability

The original contributions presented in this study are included in the article/Supplementary Materials. Further inquiries can be directed to the corresponding author.

## Supplementary Materials

The following supporting information can be downloaded at: https://www.mdpi.com/article/10.3390/healthcare14101343/s1, Table S1: CHEERS 2022 checklist; Table S2: Study characteristics of 13 studies used to estimate MASLD prevalence; Table S3: Cost-utility of three strategies over 1-year and 5-year time horizons.

## Abbreviations

The following abbreviations are used in this manuscript:

MASLD	Metabolic Dysfunction-Associated Steatotic Liver Disease
NAFLD	Non-Alcoholic Fatty Liver Disease
CAP	Controlled Attenuation Parameter
MRI-PDFF	Magnetic Resonance Imaging-Proton Density Fat Fraction
WHtR	Waist-to-Height Ratio
WTP	Willingness-To-Pay
CNSSCH	Chinese National Survey on Students Constitution and Health
NHANES	National Health and Nutrition Examination Survey
TONIC	Treatment of NAFLD in Children
TG	triglycerides
HDL	High-Density Lipoprotein
FPG	Fasting Plasma Glucose
QALYs	Quality-Adjusted Life Years
GDP	Gross Domestic Product
PSA	Probabilistic sensitivity analysis
EASL	European Association for the Study of the Liver
APASL	Asian Pacific Association for the Study of the Liver
AASLD	American Association for the Study of Liver Diseases
