# Supplementary material for: A Cost–Utility Analysis of Two-Stage Screening Strategies Based on Waist-to-Height Ratio for Pediatric Metabolic Dysfunction-Associated Steatotic Liver Disease (MASLD) in China
Source: Healthcare (Basel). 2026 May 14;14(10):1343. doi: 10.3390/healthcare14101343 (PMC13205207; doi:10.3390/healthcare14101343)
Supplement: Supplementary file 1 [file healthcare-14-01343-s001.zip › healthcare-4172286-supplementary.pdf]

Table S1 CHEERS 2022 checklist

Topic	No.	Item	Location where item is reported
**TITLE**			
**Title**	1	Identify the study as an economic evaluation and specify the interventions being compared.	Title, Page 1
**ABSTRACT**			
Abstract	2	Provide a structured summary that highlights context, key methods, results, and alternative analyses.	Summary, Page 2-3
**INTRODUCTION**			
**Background and objectives**	3	Give the context for the study, the study question, and its practical relevance for decision making in policy or practice.	Background, Paragraph 1-3
**METHODS**			
**Health economic analysis plan**	4	Indicate whether a health economic analysis plan was developed and where available.	Not reported
**Study population**	5	Describe characteristics of the study population (such as age range, demographics, socioeconomic, or clinical characteristics).	Methods, Paragraph 1
**Setting and location**	6	Provide relevant contextual information that may influence findings.	Methods, Paragraph 1
**Comparators**	7	Describe the interventions or strategies being compared and why chosen.	Methods, Paragraph 2
**Perspective**	8	State the perspective(s) adopted by the study and why chosen.	Methods, Paragraph 1
**Time horizon**	9	State the time horizon for the study and why appropriate.	Methods, Paragraph 3
**Discount rate**	10	Report the discount rate(s) and reason chosen.	Methods, Paragraph 10
**Selection of outcomes**	11	Describe what outcomes were used as the measure(s) of benefit(s) and harm(s).	Methods, Paragraph 7, 8

Topic	No.	Item	Location where item is reported
**Measurement of outcomes**	12	Describe how outcomes used to capture benefit(s) and harm(s) were measured.	Methods, Paragraph 7, 8
**Valuation of outcomes**	13	Describe the population and methods used to measure and value outcomes.	Methods, Paragraph 7, 8
**Measurement and valuation of resources and costs**	14	Describe how costs were valued.	Methods, Paragraph 7
**Currency, price date, and conversion**	15	Report the dates of the estimated resource quantities and unit costs, plus the currency and year of conversion.	Methods, Paragraph 9
**Rationale and description of model**	16	If modelling is used, describe in detail and why used. Report if the model is publicly available and where it can be accessed.	Methods, Paragraph 2-5
**Analytics and assumptions**	17	Describe any methods for analysing or statistically transforming data, any extrapolation methods, and approaches for validating any model used.	Methods, Paragraph 8-10
**Characterising heterogeneity**	18	Describe any methods used for estimating how the results of the study vary for subgroups.	Not reported
**Characterising distributional effects**	19	Describe how impacts are distributed across different individuals or adjustments made to reflect priority populations.	Methods, Paragraph 11-12
**Characterising uncertainty**	20	Describe methods to characterise any sources of uncertainty in the analysis.	Methods, Paragraph 11-12
**Approach to engagement with patients and others affected by the study**	21	Describe any approaches to engage patients or service recipients, the general public, communities, or stakeholders (such as clinicians or payers) in the design of the study.	Not applicable
RESULTS			
**Study parameters**	22	Report all analytic inputs (such as values, ranges, references) including uncertainty or distributional assumptions.	Table 1-3, Appendix table S3
**Summary of main results**	23	Report the mean values for the main categories of costs and outcomes of interest and summarise them in the most appropriate overall measure.	Results, Paragraph 1

Topic	No.	Item	Location where item is reported
**Effect of uncertainty**	24	Describe how uncertainty about analytic judgments, inputs, or projections affect findings. Report the effect of choice of discount rate and time horizon, if applicable.	Results, Paragraph 2-4
**Effect of engagement with patients and others affected by the study**	25	Report on any difference patient/service recipient, general public, community, or stakeholder involvement made to the approach or findings of the study	Not applicable
DISCUSSION			
**Study findings, limitations, generalisability, and current knowledge**	26	Report key findings, limitations, ethical or equity considerations not captured, and how these could affect patients, policy, or practice.	Discussion
**OTHER RELEVENT INFORMATION**			
**Source of funding**	27	Describe how the study was funded and any role of the funder in the identification, design, conduct, and reporting of the analysis	End of manuscript
**Conflicts of interest**	28	Report authors conflicts of interest according to journal or International Committee of Medical Journal Editors requirements.	End of manuscript

Table S2 Study characteristics of 13 studies used to estimate MASLD prevalence

First author	Publication year	Province	Diagnose	Sample size	Average age / median age (year)	Proportion of girls
Huang S. [1]	2013	Taiwan	Ultrasound	219	9.0	36.1
Lin G. [2]	2022	Liaoning	Fibroscan	1301	13.3	73.5
Liu J. [3]	2015	Fujian	Ultrasound	717	20.5	45.0
Liu Y. [4]	2024	Beijing, Zhejiang	Ultrasound & Fibroscan	1018	10.0	44.0
Meng L. [5]	2011	Beijing	Ultrasound	1449	12.0	44.2
Rong Y. [6]	2018	Shaanxi	Ultrasound	4141	18.6	49.8
Shi Z. [7]	2016	Jiangsu	ALT & overweight	19162	18.1	49.4
Wan Y. [8]	2007	Shanghai	Ultrasound	1180	9.0	51.5
Yang S. [9]	2020	Zhejiang	Ultrasound	7759	9.1	26.6
Yi X. [10]	2024	Beijing	Ultrasound	307	12.2	49.9
Zeng J. [11]	2023	Shanghai	Fibroscan	848	8.0	49.2
Zhang X. [12]	2015	Shanghai, Jiangsu, Zhejiang	Ultrasound	7229	12.3	49.0
Zhang X. [13]	2024	Liaoning	Fibroscan	462	9.9	53.9

Note: After accounting for the obesity prevalence from CNSSCH in 2019, we predicted the prevalence of MASLD among Beijing children age 6 to 9 years and 10 to 14 years were 7.0% and 9.5%, respectively.

Table S3. Cost-utility of three strategies over 1-year and 5-year time horizons

Time horizon	Strategy	Cost (million $)	Utility (QALYs)	ICUR ($/QALYs)
1 year	S1	1.93	1.51	1,282,831.7
	S2	1.92	2.09	918,326.2
	S3	3.71	2.75	1,347,598.5
	S4	0	0	Reference
5 years	S1	2.33	33.0	70,574.8
	S2	2.42	45.7	53,051.4
	S3	4.44	60.3	73,662.5
	S4	0	0	Reference

Note: S1, WHtR + Ultrasound strategy; S2, WHtR + FibroScan® strategy; S3, WHtR + MRI-PDFF strategy; S4, no screening strategy; ICUR, incremental cost-utility ratios.

Reference

1. Huang, S.C.; Yang, Y.J. Serum retinol-binding protein 4 is independently associated with pediatric NAFLD and fasting triglyceride level. *J Pediatr Gastroenterol Nutr* **2013**, *56*, 145-150, doi:10.1097/MPG.0b013e3182722aee.
2. Lin, G.; Xinhe, Z.; Haoyu, T.; Xing, J.; Dan, L.; Ningning, W.; Jing, S.; Xue, W.; Zilu, Z.; Yiling, L. Epidemiology and lifestyle survey of non-alcoholic fatty liver disease in school-age children and adolescents in Shenyang, Liaoning. *BMC Pediatr* **2022**, *22*, 286, doi:10.1186/s12887-022-03351-w.
3. Liu, J.-Q.; Zhang, Z.-J.; Wang, W.; Lin, K.-R.; Liu, G.; Jiang, C.-S.; Chen, Z.-P.; Li, H.-T.; Lin, W.-L. Risk factors for non-alcoholic fatty liver disease combined with type 2 diabetes mellitus in adolescents. *World Chinese Journal of Digestology* **2015**, *23*, 1812-1817, doi:10.11569/wcjd.v23.i11.1812.
4. Liu, Y.; Wang, Y.; Xing, Y.; Wolters, M.; Shi, D.; Zhang, P.; Dang, J.; Chen, Z.; Cai, S.; Wang, Y.; et al. Establish a noninvasive model to screen metabolic dysfunction-associated steatotic liver disease in children aged 6-14 years in China and its applications in high-obesity-risk countries and regions. *Lancet Reg Health West Pac* **2024**, *49*, 101150, doi:10.1016/j.lanwpc.2024.101150.
5. Meng, L.; Luo, N.; Mi, J. Impacts of types and degree of obesity on non-alcoholic fatty liver disease and related dyslipidemia in Chinese school-age children? *Biomed Environ Sci* **2011**, *24*, 22-30, doi:10.3967/0895-3988.2011.01.003.
6. Rong, Y.; Chun-Yan, N.; Hong-Xin, Z.; Lu, Y.; Wen, W.; Yu, T. Association of Adolescent Obesity with Nonalcoholic Fatty Liver Disease and Related Risk Factors in Xi 'an, China. *Ann Hepatol* **2018**, *17*, 85-91, doi:10.5604/01.3001.0010.7538.
7. Shi, Z.; Chen, W.; Taylor, A.; Burt, A. Elevated transaminase levels among overweight adolescents in eastern China. *Acta Paediatr* **2016**, *105*, e593-e599, doi:10.1111/apa.13521.
8. Wan, Y.P.; Xu, R.Y.; Fang, H.; Lu, L.P.; Zhang, X.M.; Cai, W. [The prevalence of non-alcoholic fatty liver disease and its related risk factors in 1180 school children in Shanghai]. *Zhonghua Gan Zang Bing Za Zhi* **2007**, *15*, 644-648.
9. Yang, S.; Zhong, J.; Ye, M.; Miao, L.; Lu, G.; Xu, C.; Xue, Z.; Zhou, X. Association between the non-HDL-cholesterol to HDL-cholesterol ratio and non-alcoholic fatty liver disease in Chinese children and adolescents: a large single-center cross-sectional study. *Lipids Health Dis* **2020**, *19*, 242, doi:10.1186/s12944-020-01421-5.
10. Yi, X.H.; Han, L.W.; Li, L.X.; Zhu, H.X.; Li, M.; Gao, S. Adipokine/hepatokines profiling of fatty liver in adolescents and young adults: cross-sectional and prospective analyses of the BCAMS study. *Hepatology international* **2025**, *19*, 143-155, doi:10.1007/s12072-024-10736-9.
11. Zeng, J.; Jin, Q.; Yang, J.; Yang, R.X.; Zhang, R.N.; Zhao, J.; Fan, J.G. Prevalence and incidence of MAFLD and associated anthropometric parameters among prepubertal children of the Shanghai Birth Cohort. *Hepatol Int* **2023**, *17*, 1416-1428, doi:10.1007/s12072-023-10574-1.
12. Zhang, X.; Wan, Y.; Zhang, S.; Lu, L.; Chen, Z.; Liu, H.; Jiang, X.; Luo, K.; Cai, W. Nonalcoholic fatty liver disease prevalence in urban school-aged children and adolescents from the Yangtze River delta region: a cross-sectional study. *Asia Pac J Clin Nutr* **2015**, *24*, 281-288, doi:10.6133/apjcn.2015.24.2.13.
13. Zhang, X.; Tian, H.; Guan, L.; Li, Y. Investigation on the epidemiology and living habits of non-alcoholic fatty liver disease among primary school students in Shenyang city. *Journal of Health Examination and Management* **2024**, *5*, 125-132.
